# Overexpression of BMI-1 Promotes Cell Growth and Resistance to Cisplatin Treatment in Osteosarcoma

**DOI:** 10.1371/journal.pone.0014648

**Published:** 2011-02-02

**Authors:** Zhihong Wu, Li Min, Dafu Chen, Dongsheng Hao, Yuanhui Duan, Guixing Qiu, Yipeng Wang

**Affiliations:** 1 Department of Orthopaedics, Peking Union Medical College Hospital, Chinese Academy of Medical Sciences and Peking Union Medical College, Beijing, China; 2 Laboratory of Bone Tissue Engineering, Beijing Research Institute of Traumatology and Orthopaedics, Beijing, China; Institute of Zoology, Chinese Academy of Sciences, China

## Abstract

**Background:**

BMI-1 is a member of the polycomb group of genes (PcGs), and it has been implicated in the development and progression of several malignancies, but its role in osteosarcoma remains to be elucidated.

**Methodology/Principal Findings:**

In the present study, we found that BMI-1 was overexpressed in different types of osteosarcomas. Downregulation of BMI-1 by lentivirus mediated RNA interference (RNAi) significantly impaired cell viability and colony formation *in vitro* and tumorigenesis *in vivo* of osteosarcoma cells. BMI-1 knockdown sensitized cells to cisplatin-induced apoptosis through inhibition of PI3K/AKT pathway. Moreover, BMI-1-depletion-induced phenotype could be rescued by forced expression of BMI-1 wobble mutant which is resistant to inhibition by the small interfering RNA (siRNA).

**Conclusions/Significance:**

These findings suggest a crucial role for BMI-1 in osteosarcoma pathogenesis.

## Introduction

Primary osteosarcoma is the most common bone tumor that predominantly develops in adolescents and young adults [1]. Even after the introduction of aggressive chemotherapy and wide excision of tumors, 30–50% of patients with initially localized disease subsequently developed recurrence, which has an extremely poor clinical outcome. Moreover, 20–30% of newly diagnosed cases presented with metastatic disease [2], [3]. Therefore, identifying the effector molecules and/or signal transduction pathways that regulate the carcinogenesis and malignant development is crucial for understanding the vulnerabilities of osteosarcoma and for isolating molecular targets that may disrupt the tumor machinery whereas protecting the integrity and function of normal tissue.

BMI-1 is a member of the polycomb group family of transcriptional regulators that was originally identified as an oncogenic partner of c-Myc in murine lymphomagenesis [4]. BMI-1 is essential for blood-cell development and the self-renewal capacity of several types of normal and cancer stem cells [5], and it is also important for cell cycle regulation, since both p16/INK4a and p14/ARF are downstream targets of BMI-1 [6], [7]. Experimental studies convincingly link BMI-1 to carcinogenesis, for instance, aberrant expression of BMI-1 has been associated with malignant tumors of the hematopoietic and lymphatic systems [8], [9], melanoma [10], neuroblastoma [11], endometrial carcinoma [12], non-small cell lung cancer [13] and breast cancer [14]. Further more, high expression of BMI-1 in a panel of epithelial cancers including nasopharyngeal was associated with poor patient outcome [15], [16]. Molecular investigations have suggested a role for BMI-1 by immortalization of mammary epithelial cells through induction of telomerase activity and regulation of cancer cells with stem-cell like properties [17], [18]. Overexpression of BMI-1 downregulates expression of p16INK4a and p14ARF, allows fibroblast immortalization, and in combination with H-ras leads to neoplastic transformation [14].

Although BMI-1 has been implicated as an oncogenic player, very little is known about the exact function of BMI-1 in osteosarcoma growth and progression. In this study we have investigated whether BMI-1 functions as an oncogene in osteosarcoma. Our findings confirm that BMI-1 is highly expressed in osteosarcoma cells, and it promotes tumor growth *in vitro* and *in vivo*. Furthermore, our data show that BMI-1 also play an important role in osteosarcoma cell migration and chemosensitivity to cisplatin-induced apoptosis.

## Results

### Overexpression of BMI-1 in osteosarcoma

**Figure 1:**
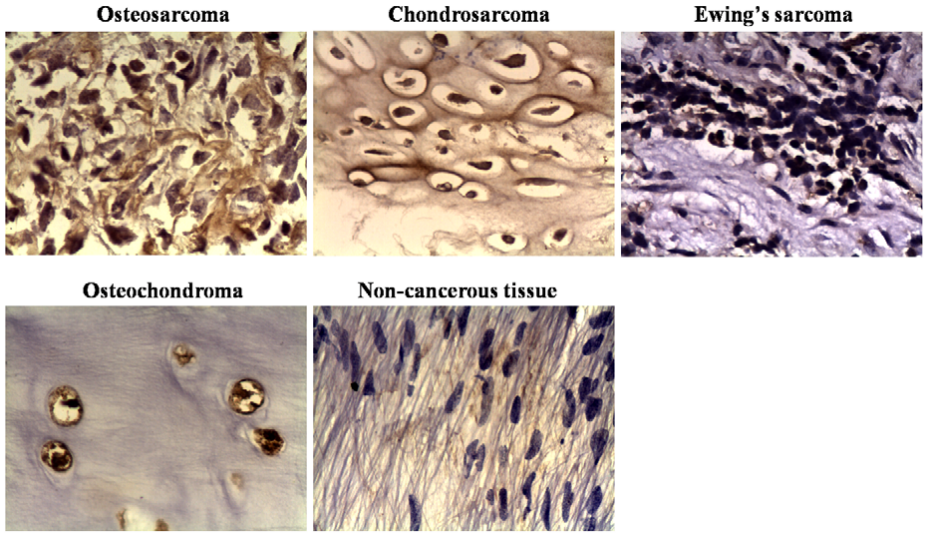
Immunohistochemical staining of BMI-1. BMI-1 immunoreactivity was localized in both the nucleus and cytoplasm. Images of positive BMI-1 staining in nucleus of osteosarcoma, osteochondroma and chondrosarcoma, and in cytoplasm of Ewing's sarcoma were shown (×400). Negative BMI-1 staining in a non-cancerous tissue sample served as control.

As elevated BMI-1 expression was found in several types of human cancers, we first determine the expression of BMI-1 protein in osteosarcoma through immunohistochemical analysis. BMI-1 expression was not detected in 10 patients with noncancerous bone tissues. In contrast, positive expression of BMI-1 was observed 18/32 in osteosarcoma, 9/22 in chondrosarcoma, 3/9 in Ewing's sarcoma and 5/27 in osteochondroma (a benign neoplasm) (Figure 1). These data indicated that BMI-1 is ubiquitously upregulated in some types of osteosarcoma. Statistical analysis indicated that positive expression of BMI-1 in benign and malignant bone tumors was significantly different, but elevated expression of BMI-1 was not correlated with any clinicopathologic parameters in the available 90 specimens.

### BMI-1 knockdown inhibits osteosarcoma cell growth both *in vitro* and *in vivo*

**Figure 2:**
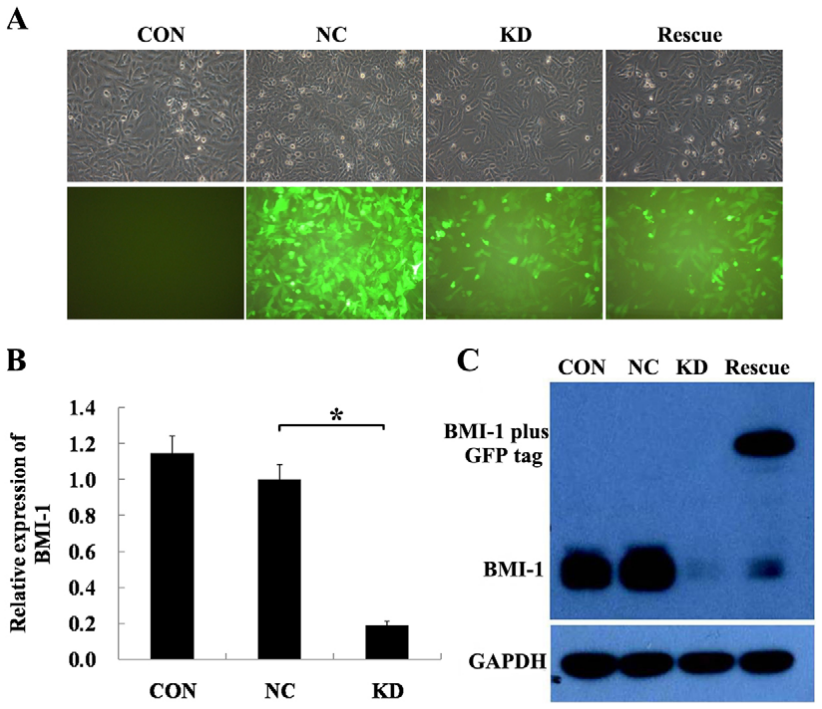
Targeted depletion of BMI-1 through lentivirus-mediated siRNA. (A) Representetive graphs of SAOS-2 cells infected with indicated lentivirus at MOI of 10 were shown (×400). Following infection of cells with indicated lentivirus for 5 days, BMI-1 mRNA levels were measured with real-time PCR (B), and protein levels were detected by Western blot analysis (C). *: *P*<0.01, compared to control cells. Con: non-infected, NC: non-silencing, KD: BMI-1 knock down, Rescue: BMI-1 wobble mutant.

To suppress BMI-1 expression in osteosarcoma cells, short harpin RNA (shRNA) targeting BMI-1 gene was designed and inserted into the recombinant lentivirus plasmid. To verify that the effects of RNA interference (RNAi) are specific, we prepared a BMI-1 construct bearing triple-point mutation in the 19-bp sequence that served as target for small interfering RNA (siRNA)-mediated knockdown. The mutation conserved the amino acid sequence of the BMI-1 protein but rendered its expression insensitive to inhibition by the siRNA. Effeciency of lentivirus infection was more than 90% as evidenced by GFP expression 3 days after infection (Figure 2A). BMI-1 mRNA expression was then measured with real-time PCR. As shown in Figure 2B, endogenous BMI-1 mRNA was obviously reduced in BMI-1 knockdown group. We further determined the suppression effect by measuring BMI-1 protein levels using immunoblot. It was demonstrated that BMI-1 protein level was significantly downregulated following BMI-1 knockdown treatment (Figure 2C).

**Figure 3:**
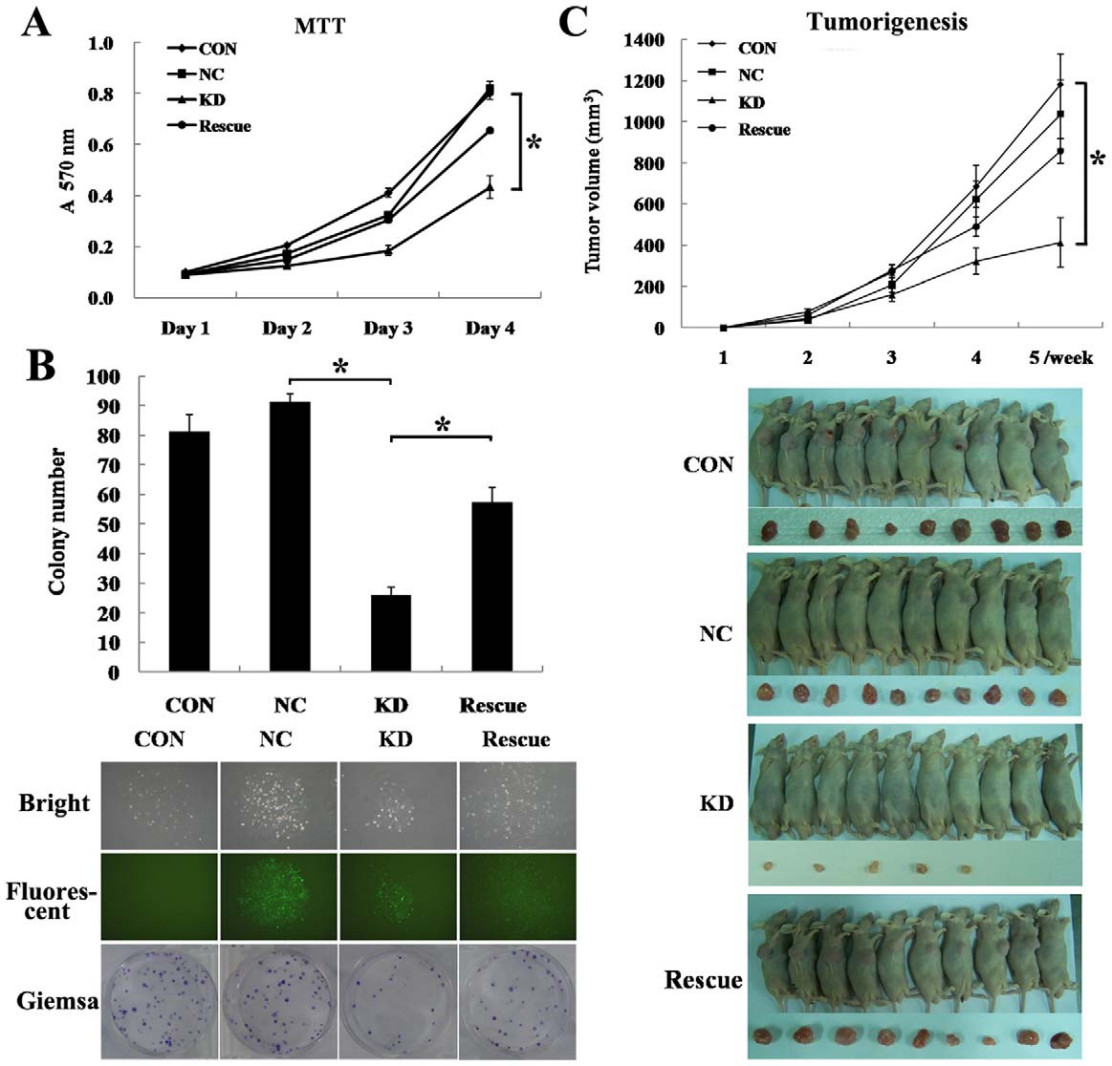
Effects of BMI-1 siRNA on the *in vitro* proliferation, colony formation, and *in vivo* tumorigenicity of osteosarcoma cells. (A) The monolayer growth rates of SAOS-2 cells from different groups were determined by MTT assay. (B) Downregulation of BMI-1 inhibited colony-forming ability. Photographs of plates and representative colonies were shown. Histogram was the average number of colonies in each plate. (C) Downregulation of BMI-1 reduced the tumorigenicity of SAOS-2 cells. The change of tumor volume during 5 weeks of inoculation was measured. Representative pictures of tumor bearing mice and tumors were shown (n = 10). Values represent the mean (standard error of the mean) from at least three separate experiments. *: *P*<0.01, compared to control cells. Con: non-infected, NC: non-silencing, KD: BMI-1 knock down, Rescue: BMI-1 wobble mutant.

To elucidate the role of BMI-1 in osteosarcoma proliferation and tumorigenesis, the growth of each lentivirus infected SAOS-2 cells were first examined by MTT assay. Figure 3A showed that the growth curves for BMI-1 knockdown cells were significantly lower than those for control cells and BMI-1 rescued cells for 4 days of incubation. Furthermore, colony formation assay in monolayer culture showed that the number of surviving colonies of BMI-1 knockdown cells was markedly decreased compared to those of control cells, suggesting that BMI-1 expression is detrimental to the colony formation of osteosarcoma cells (Figure 3B). Next, *in vivo* subcutaneous tumor formative assay was adopted to examine the tumorigenesis of SAOS-2 cells in nude mice. Compared to control cells expressing non-silencing small interfering RNA (siRNA), the injection of BMI-1 knockdown cells led to a significantly decrease in tumor volume and size. Conversely, BMI-1 rescued cells partially restored the proliferative ability in nude mice (Figure 3C). Both *in vitro* and *in vivo* assays suggested that BMI-1 had the potential to inhibit proliferation, colony formaton, and tumorigenicity of osteosarcoma.

### Downregulation of BMI-1 inhibits osteosarcoma cell migration

**Figure 4:**
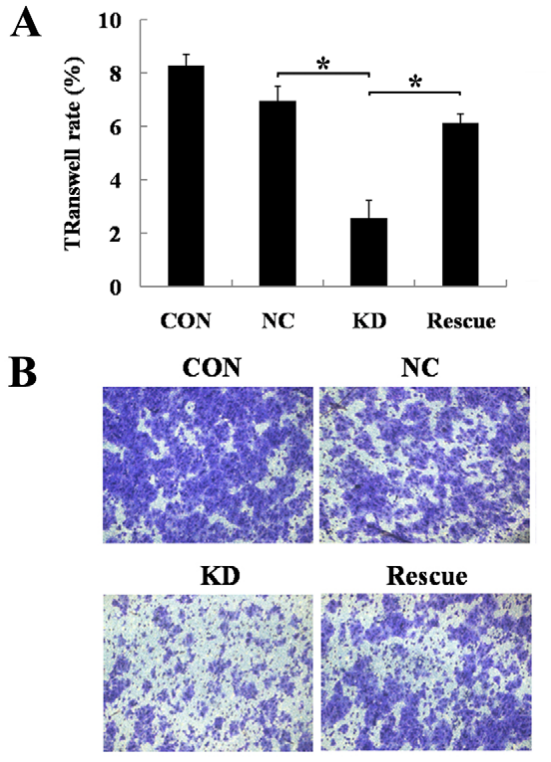
Downregulation of BMI-1 suppresses osteosarcoma cell migration. (A) Statistical plots of haptotactic migration assay. Columns, mean of three individual experiments; bars, SD;*: *P*<0.01, compared to indicated cells. (B) Representative photos of haptotactic migration assay. Con: non-infected, NC: non-silencing, KD: BMI-1 knock down, Rescue: BMI-1 wobble mutant.

We also examined the role of BMI-1 in osteosarcoma cell migration. SAOS-2 cells treated with BMI-1 siRNA-expressing lentivirus demonstrated decreased ability to migrate through 8-µm-pore-size membranes that were not coated with Matrigel (Figure 4). More importantly, when BMI-1 knockdown cells were further infected with the lentivirus encoding a BMI-1 wobble mutant, cell migration was substantially rescued (*P*<0.05).

### Knockdown of BMI-1 sensitized osteosarcoma cell to cisplatin treatment

**Figure 5:**
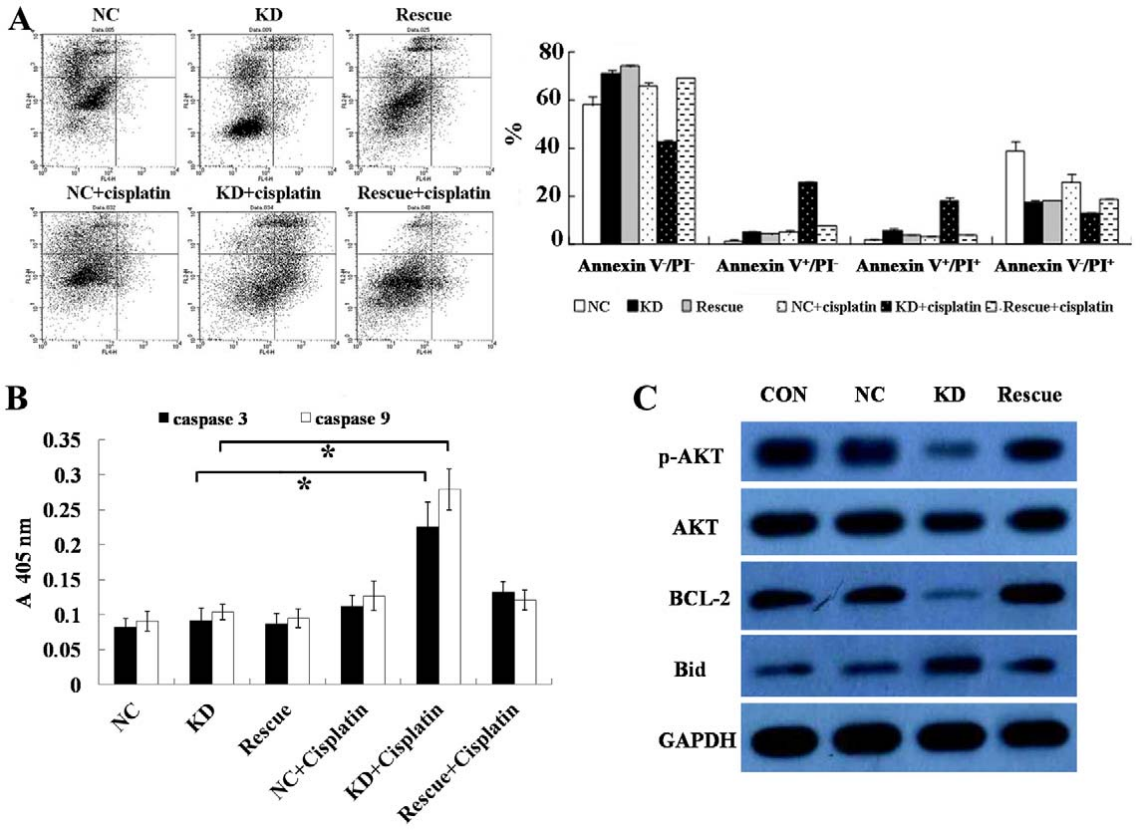
BMI-1 depletion sensitized cells to cisplatin treatment through PI3K/AKT pathway. After infection, SAOS-2 cells were treated with 10 µg/ml cisplatin for 24 h and subjected to Annexin V-PI Apoptosis analysis (A) and measurement of caspase-3 and caspase-9 activities (B). (C) Western blot analysis of p-AKT, AKT, BCL-2 and Bid protein. *: *P*<0.01, compared to control cells. Con: non-infected, NC: non-silencing, KD: BMI-1 knock down, Rescue: BMI-1 wobble mutant, Annexin V^−^/PI^−^: viable cells, Annexin V^+^/PI^−^: cells in early apoptosis, Annexin V^+^/PI^+^: cells in late apoptosis, Annexin V^−^/PI^+^: cells in necrosis.

It has been reported that knockdown of BMI-1 makes nasopharyngeal carcinoma cells more sensitive to 5-FU treatment and that depletion of BMI-1 enhances 5-FU-induced apoptosis [19]. These observations prompted us to investigate the possibility of the combination of cisplatin treatment and BMI-1 depletion as a clinical strategy for osteosarcoma chemotherapy. As shown in Figure 5A, total percent of apoptotic BMI-1 knockdown cells (cells in early and late apoptosis) was about double that of the control and parental cells in the assessment by Annexin V-FITC/PI (propidium iodide) staining. However, the addition of cisplatin significantly increased apoptosis in BMI-1 knockdown cells, suggesting that combining BMI-1 inhibition with cisplatin enhanced the incidence of apoptosis.

We subsequently explore the possible molecular mechanism by which BMI-1 protected osteosarcoma cells from cisplatin induced apoptosis. Caspases are important regulators of apoptosis. Therefore, the involvement of caspase-3 and caspase-9 in cisplatin-induced apoptosis was investigated. In the BMI-1 knockdown cells, after treatment with cisplatin (10 µg/ml), caspase-3 and caspase-9 activities were observed to be increased by 2.4-fold and 2.7-fold, respectively (Figure 5B).

Further more, expression levels of total-AKT, phospho-AKT (p-AKT), Bid and BCL-2 were examined in BMI-1 knockdown cells, BMI-1 rescued cells and control cells. Immunoblot analysis showed that the knockdown of endogenous BMI-1 led to significant reduction in the levels of phospho-AKT and BCL-2, whereas pro-apoptotic protein Bid was elevated in BMI-1 knockdown cells. Nevertheless, wobble mutant of BMI-1 rescued the expression of phospho-AKT, BCL-2 and Bid protein in SAOS-2 knockdown cells (Figure 5C).

## Discussion

In the present study, we found that BMI-1 was highly expressed in malignant osteosarcoma, and it is essential for cancer cell proliferation, migration and *in vivo* tumorigenicity. We demonstrated for the first time that the knockdown of endogenous BMI-1 expression contributed to sensitizing osteosarcoma cells to the anticancer drug cisplatin by increasing apoptosis.

Recently, it is reported that osteosarcoma might originate from somatic stem cells [20]. Given that BMI-1 is upregulated in most stem cells and its expression is vital for the self-renewal of normal and tumorigenic stem cells [21], it is possible that the high level of BMI-1 expression we observed in osteosarcoma cells is an inherent feature of their cellular origin. Recently, Douglas *et al.* also found that BMI-1 is highly expressed in Ewing sarcoma family of tumors and that it regulates sarcoma cell adhesion and growth in nude mice [22]. In addition their expression profiling studies following BMI-1 knockdown revealed that part of downstream target genes of BMI-1 were involved in cell-cell and cell-matrix adhesion. However, they observed that BMI-1 does not significantly affect proliferation or survival, suggesting that this property of BMI-1 is limited to certain type of osteosarcoma cells.

Due to the therapeutic advantages, such as high efficiency, mild side effects and easy administration, cisplatin is still one of the most common used agents in chemotherapy. However, resistance to this drug is also often observed, and therefore enhancing the sensitization of cancer cells to cisplatin-induced apoptosis has become an important strategy for chemotherapy. We found that the depletion of BMI-1 in SAOS-2 cells, in which BMI-1 is highly expressed, resulted in an increased sensitivity of these cells to cisplatin. Pro-caspase-3, an effector caspase of apoptosis, is one of the effector pro-caspases activated by caspase-9 [23]. Measurement of caspase-3 and caspase-9 activities further confirmed that silencing BMI-1 expression could enhance cisplatin-induced apoptosis, further explaining clearly the signaling pathway of cisplatin-induced apoptosis in osteosarcoma.

It is well known that p53 induces apoptosis in response to DNA damage, and SAOS-2 cells have a wild-type p53. Therefore we hypothized that p53 might be responsible for increased apoptosis after cisplatin treatment in BMI-1 knockdown cells. However, mRNA and protein levels of p53 and its effecter p21 were downregulated upon BMI-1 knockdown (data not shown). We assumed that the apoptosis in the SAOS-2 cells reported herein was p53-independent. Activation of the PI3K/AKT pathway enhances resistance to apoptosis is observed in a wide variety of cancers [24], [25]. In the present study, inhibition of the PI3K/AKT pathway after BMI-1 knockdown was found to play a role in the sensitivity of SAOS-2 cells to cispatin treatment. The ratio of the anti-apoptotic to pro-apoptotic proteins is important in determining cell apoptosis and survival after DNA damage. We also found decreased BCL-2 levels and Bid accumulation in BMI-1 knockdown cells, and BCL-2 and Bid levels were attractively rescued with overexpression of BMI-1, indicating that BMI-1 could regulate the ratio of BCL-2 to Bid in SAOS-2 cells.

In summary, our results demonstrated that BMI-1 functions as an oncogene in osteosarcoma and that it promotes tumorigenicity and resistance to chemotherapy. Our findings suggest that the combination of cisplatin treatment and BMI-1 depletion could be a therapeutic strategy for osteosarcoma.

## Materials and Methods

### Tissue specimens

Ninety patients who were histologically confirmed as having osteosarcoma, chondrosarcoma, Ewing sarcoma, osteochondroma, and normal bone tissues were enrolled Peking Union Medical College Hospital, China. The clinicopathologic variables such as gender, age, the histologic type and the status of the resection margin were retrospectively reviewed on the basis of the medical records. All patients received no treatment before surgery. The samples were used with the written informed consent from patient and the approval of the ethic committee of Peking Union Medical College Hospital, China.

### Cell culture

Human embryonic kidney 293T (HEK293T) cell line and the osteosarcoma cell line (SAOS-2) were obtained from the American Type Culture Collection (ATCC, Rockville, MD, USA). Cells were cultured in Dulbecco's modified Eagle's medium (Gibco RL, Grand Island, NY, USA) supplemented with 10% fetal bovine serum, 100 U/ml penicillin, and 100 µg/ml streptomycin.

### Construction of recombinant Lentivirus and gene silencing

The siRNA sequence for BMI-1 (5′-CAGATTGGATCGGAAAGTA-3′) was selected after screening to validate potential siRNAs. Non-silencing siRNA (5′-TTCTCCGAACGTGTCACGT-3′) was used as control. ShRNAs, corresponding to siRNA sequences for BMI-1 and non-silencing, were then generated, respectively. These were synthesized as a 21-nt inverse repeat separated by a 9-nt loop for each sequence and inserted downstream of the U6 promoter in the lentiviral vector pGCL-GFP (GeneChem Co., Ltd., Shanghai, China). Lentiviruses were generated by triple transfection of 80% confluent HEK293T cells with modified pGCL-GFP plasmid and pHelper 1.0 and pHelper 2.0 helper plasmids (GeneChem Co., Ltd., Shanghai, China) using Lipofectamine 2000 (Invitrogen, Carlsbad, CA, USA). Lentiviruses were harvested in serum-free medium after 3 days, filtered and concentrated in primed Centricon Plus-20 filter devices (Millipore, Billerica, MA, USA). BMI-1 wobble mutant preserving the same amino acid sequence, but containing triple-point mutations in the target nucleotide sequence and therefore resistant to BMI-1 siRNA, was produced by GeneChem Co., Ltd. (Shanghai, China).

### RNA extraction and real-time PCR

Total RNA was prepared using Trizol reagent (Gibco RL, Grand Island, NY, USA) according to the manufacturer's instruction. Five µg of total RNA was used to synthesize the first strand of cDNA using SuperScript II RT 200 U/µl (Invitrogen, Carlsbad, CA, USA). BMI-1 mRNA expression was evaluated by real-time PCR on an ABI Prism® 7300 (Applied Biosystems, Foster City, CA, USA) with SYBR Green PCR core reagents. β-actin was applied as the input reference. The following primers were used: BMI-1: 5′-TGGCTCGCATTCATTTTCTG-3′ as forward and 5′-AGTAGTGGTCTGGTCTTGTG-3′ as reverse; β-actin: 5′-GGCGGCACCACCATGTACCCT-3′ as forward and 5′-AGGGGCCGGACTCGTCATACT-3′ as reverse. Results are presented as C_T_ values, defined as the threshold PCR cycle number at which an amplified product is first detected. The average C_T_ was calculated for both BMI-1 and β-actin, and ΔC_T_ was determined as the mean of the triplicate C_T_ values for BMI-1 minus the mean of the triplicate C_T_ values for β-actin.

### Immunohistochemical staining

The slides were deparafinized, rehydrated, then immersed in 3% hydrogen peroxide solution for 10 min, heated in citrate buffer, pH 6.0, at 95°C for 25 min, cooled at room temperature for 60 min. The slides were blocked by 10% normal goat serum at 37°C for 30 min, and then incubated with rabbit polyclonal antibody against BMI-1(1: 200, ProteinTech Group, Chicago, IL, USA) for overnight at 4°C. After washing with PBS, the slides were incubated with biotionylated second antibody (diluted 1: 100) for 30 min at 37°C, followed by streptavidin-peroxidase (1: 100 dilution) incubation at 37°C for 30 min. Immunolabeling was visualized with a mixture of DAB solution. Counterstaining was carried out with hematoxylin. Samples were scored positive when more than 10% of the cells reacted with the anti-BMI-1 antibody and presented cytoplasm staining.

### Western blot analysis

Proteins were separated by SDS–PAGE, transferred to polyvinylidene difluoride (PVDF) membranes (Millipore, Bedford, MA, USA). Blots were blocked and then probed with antibodies against BMI-1(1: 500 dilution; Santa Cruz Biotechnology, Santa Cruz, CA, USA), AKT (1: 1000 dilution; Santa Cruz Biotechnology, Santa Cruz, CA, USA), phospho-AKT (Ser473; 1: 400 dilution; Santa Cruz Biotechnology, Santa Cruz, CA, USA), Bcl-2 (1: 500 dilution; Cell Signaling Technology Inc., Beverly, Massachusetts, USA), Bid (1: 500 dilution; Cell Signaling Technology Inc., Beverly, Massachusetts, USA), Mouse anti-GAPDH, (1: 5000 dilution; Santa Cruz Biotechnology, Santa Cruz, CA, USA). After washing, the blots were incubated with horseradish peroxidase-conjugated secondary antibodies and visualized by super ECL detection reagent (Applygen, Beijing, China).

### Cell survival analysis

Briefly, cells from different groups were seeded at an initial density of 5×10^4^ cells/ml in 96-well plates for 24 h. 3-(4, 5-Dimethylthiazol-2-yl)-2, 5-diphenyltetrazolium bromide (MTT) was added into each well at a final concentration of 5 mg/ml for 4 h. DMSO was then added to stop the reaction and measured with an ELISA reader (Bio-Rad, Hercules, CA, USA) at a wavelength of 570 nm. Viability of cells was expressed relative to theoretical absorbance (A).

### Colony formation assay and tumor formation in nude mice

To assay monolayer colony formation, 200 infected cells were plated in 6-well plates. After 2 weeks, cells were fixed with methanol and stained with Giemsa. The number of colonies was counted.

To assay tumor formation in nude mice, cloned pools of BMI-1 knockdown cells, scrambled RNAi cells, BMI-1 rescued cells, and parental SAOS-2 cells were trypsinized, counted, and resuspended in 1×PBS. One hundred microlitre of 1×PBS containing 10^6^ cells was then infected into 4-to 5-week-old female nude mice which were purchased from Weitonglihua Company (Beijing, China). The mice were examined for subcutaneous tumor growth and sacrificed 5 weeks after injection. Animal study protocol was reviewed and approved by the Institutional Animal Care and Use Committee of Peking Union Medical College Hospital, China [the permit number: SCXK (Jing) 2007-0001].

### Migration assay

For haptotactic cell migration assay, 1×10^4^ cells from different groups were seeded on a fibronectin-coated polycarbonate membrane insert (6.5 mm in diameter with 8.0 µm pores) in a transwell apparatus (Costar, Cambridge, MA, USA) and cultured in DMEM. FBS was added to the lower chamber. After incubation for 12 h at 37°C in a CO_2_ incubator, the insert was washed with PBS, and cells on the top surface of the insert were removed by wiping with a cotton swab. Cells that migrated to the bottom surface of the insert were fixed with methanol and stained by Giemsa and then subjected to microscopic inspection. Cells were counted based on five field digital images taken randomly at ×200.

### Flow cytometry assay

After lentivirus infection and cisplatin (10 µg/ml) treatment, cells in each well were harvested and cell apoptosis was determined by Annexin V-FITC/PI staining method. Tests were performed in triplicate for each sample, and analyses were performed by FAC-Scan flow cytometer (Becton Dickinson, San Jose, CA, USA) in accordance with the manufacturer's guidelines.

### Measurement of caspase-3 and caspase-9 activities

The activation of caspase-3 and caspase-9 were determined in SAOS-2 cells with the colorimetric kit (Nanjing kaiji Bio-Tek Corporation, China). Cells (3×10^6^) were harvested and washed twice with PBS. After the cells were lysed, 50 µl of 2x reaction buffer was added followed by the additional 5 µl of caspase-3 or caspase-9 substrate and incubated at 37°C for 4 h. The plate was then read with an ELISA reader (Bio-Rad, Hercules, CA, USA) at 405 nm.

### Statistical analysis

All statistical analyses were performed using SPSS13.0 software. The differences between groups were compared using Student's t-test, and data were expressed as mean ± SD. Statistical difference was accepted at *P*<0.05.
